# A Comprehensive Analysis to Elucidate the Effects of Spraying Mineral Elements on the Accumulation of Flavonoids in *Epimedium sagittatum* during the Harvesting Period

**DOI:** 10.3390/metabo13020294

**Published:** 2023-02-16

**Authors:** Linlin Yang, Fei Zhang, Yueci Yan, Xupeng Gu, Shengwei Zhou, Xiuhong Su, Baoyu Ji, Hua Zhong, Chengming Dong

**Affiliations:** 1 Henan Provincial Ecological Planting Engineering Technology Research Center of Daodi Herbs, School of Pharmacy, Henan University of Chinese Medicine, Zhengzhou 450046, China; 2 Co-Construction Collaborative Innovation Centre for Chinese Medicine and Respiratory Diseases by Henan & Education Ministry of PR China, Henan University of Chinese Medicine, Zhengzhou 450046, China; 3 Rural Agriculture Bureau of Pingyu County, Zhumadian 463499, China

**Keywords:** *Epimedium sagittatum*, flavonoids, icariin, FT-IR, metabolomic

## Abstract

The harvesting period is a critical period for the accumulation of flavonoids in the leaves of the important medicinal plant *Epimedium sagittatum*. In this study, we conducted an experiment on *E. sagittatum* leaves sprayed with mineral elements with the aim of improving the quality of the herbal leafage during the harvesting period. We elucidated the changes in flavonoids (icariin, epimedin A, epimedin B, and epimedin C) in *E. sagittatum* leaves. The sum of main flavonoids content reached a maximum (11.74%) at 20 days after the high-concentration Fe^2+^ (2500 mg·L^−1^) treatment. We analyzed the FT-IR spectra characteristics of *E. sagittatum* leaf samples using the FT-IR technique, and constructed an OPLS-DA model and identified characteristic peaks to achieve differentiated identification of *E. sagittatum*. Further, widely untargeted metabolomic analysis identified different classes of metabolites. As the most important characteristic flavonoids, the relative contents of icariin, icaritin, icariside I, and icariside II were found to be up-regulated by high-Fe^2+^ treatment. Our experimental results demonstrate that high-concentration Fe^2+^ treatment is an effective measure to increase the flavonoids content in *E. sagittatum* leaves during the harvesting period, which can provide a scientific basis for the improvement of *E. sagittatum* leaf cultivation agronomic measures.

## 1. Introduction

*Epimedium folium* (yin yang huo in Chinese), a yang-invigorating herb, is a commonly used herbal leafage (a class of Chinese herbs used as medicine) that has been used for over 2000 years in China [1]. *Epimedium folium* is derived from the dried leaves of *Epimedium sagittatum*, *E. brevicomu*, *E. pubescens*, and *E. koreanum* of the genus Berberidaceae, and is one of the most widely used Chinese herbs in the Chinese Pharmacopoeia [2]. There are about 60 species of *Epimedium* in the family Berberaceae, of which more than 50 species are concentrated in southwest and central China [3,4], and although they are not all included in the Chinese Pharmacopoeia, they are still used to a small extent in folklore. Modern pharmacological studies have shown that *Epimedium folium* plays an active role in the treatment of hypertension, coronary heart disease, osteoporosis, menopausal syndrome, impotence, hemiplegia, and numbness of the limbs [5,6,7]. Against the background of advocating the development of Traditional Chinese Medicine, as the use of *Epimedium folium* rises year by year, its market demand also gradually expands, and the current wild-harvested *Epimedium folium* (the main source) can no longer meet the huge consumer demand. Thus, seeking effective measures to improve the quality of *Epimedium folium* under artificial cultivation conditions and expanding *Epimedium folium* production is one of the paths that must be developed in the agricultural production of traditional Chinese medicine at present.

*E. sagittatum* is a medicinal plant, the leaves of which are the main medicinal part [8], and its main bioactive components are flavonoids (mainly icariin, epimedin A, epimedin B, and epimedin C), commonly used for kidney yang deficiency, impotence and spermatorrhea, tendon impotence, and other symptoms [9]. In particular, the main bioactive component is icariin, which has the biological activities of multi-target anti-inflammatory and anti-tumor immune regulation [10], and its safety and efficacy have been demonstrated in phase I, II, and III clinical trials in advanced hepatocellular carcinoma cells in China [11,12]. It has thus attracted much attention. With the launch of a new drug (icaritin soft capsule) in China, the demand for *E. sagittatum* is further increasing. Therefore, under the current situation that the wild resources of *E. sagittatum* are gradually being depleted, how to increase flavonoids content, as well as plant biomass under artificial cultivation, is one of the urgent problems that needs to be solved. Determining the appropriate time for harvesting in agricultural production is particularly important for medicinal plants (herbal medicines) [13]. This is because the period of highest bioactive component content in medicinal plants often does not coincide with the period of greatest biomass. For example, the traditional Chinese medicine *Artemisae scopariae* Herba is derived from the dried above-ground part of *Artemisae scopariae* and *A. capillaris*, which requires that *A. scopariae* and *A. capillaris* must be harvested in the spring, when they are a young seedling, or in the fall, when the flower buds have grown to the point of flower removal for use in medicine [14]. *Lippia alba* is widely used as a seasoning and traditional medicine in Brazil; in it, phenylpropanoids are predominant in winter, while flavonoids are predominant in summer, and other major secondary metabolites’ contents vary with the month [15]. For *E. sagittatum*, which is used as a herbal leafage, the main bioactive components, flavonoids, tend to decrease gradually as the leaves mature [16], so the contents of the bioactive components that are sought do not reach the maximum at the time of harvesting in autumn. For this reason, the search for effective measures to increase the flavonoids content of *E. sagittatum* during the autumn harvesting period is practical and effective in relation to the improvement of artificial cultivation techniques.

In the process of cultivating medicinal plants, fertilizers provide essential nutrients, and improve soil properties and soil fertility [17]. Foliar fertilizer can be applied directly to the foliage of medicinal plants, and can thus be used as a supplement to soil fertilizer and has the characteristics of fast fertilization effects, high nutrient utilization rate, and low environmental pollution [18]. A study by Bayram et al. on *Pistacia lentiscus* var. *chia* (an important medicinal aromatic plant) showed that the foliar spraying of Zn^2+^ increased the β-myrcene, germacrene-D, and α-pinene contents, particularly at 100% leafing [19]. Studies on red rice (a *Oryza sativa* with functional food potential) found that spraying with Fe^2+^ could promote the accumulation of procyanidin in grains by up-regulating the expressions of *F3H* and *ANS* genes [20]. Spraying Cu^2+^ on *Mentha spicata* is conducive to the biosynthesis of its major volatile oil components, resulting in significant changes in the yield and components of volatile oil [21]. For artificially cultivated *E. sagittatum*, the biomass of its leaves stabilizes during the harvesting period of autumn, but it is not clear whether its flavonoid content can be increased by spraying with mineral elements. Therefore, conducting relevant studies to elucidate the effects of spraying mineral elements on the flavonoid contents of *E. sagittatum* leaves is of practical importance to developing a simple operational technique that can improve the quality of medicinal herbs.

In order to elucidate the effects of spraying with mineral elements on the flavonoid’s accumulation of *E. sagittatum* leaves in a comprehensive analysis, we conducted an experiment on the changes of flavonoid contents in *E. sagittatum* leaves after spraying with different concentrations of mineral elements (Fe^2+^ Cu^2+^ and Zn^2+^), with the aim of improving the quality of the herbal leafage during the harvesting period. We analyzed the changes in flavonoid (icariin, epimedin A, epimedin B, and epimedin C) contents after 10 and 20 days of spraying with mineral elements, using the high-performance liquid chromatography (HPLC) technique as the quantitative analysis, and outlined the characteristics of the Fourier transform infrared (FT-IR) spectra of different *E. sagittatum* samples as the qualitative analysis. In addition, we further analyzed the effects of spraying high concentrations of Fe^2+^ on the synthesis of secondary metabolites in *E. sagittatum* leaves during harvesting, using widely untargeted metabolomic techniques. This study can provide a scientific basis for the improvement of agronomic cultivation measures during the harvesting period of *E. sagittatum*.

## 2. Materials and Methods

### 2.1. Experimental Materials and Treatment Methods

**Figure 1:**
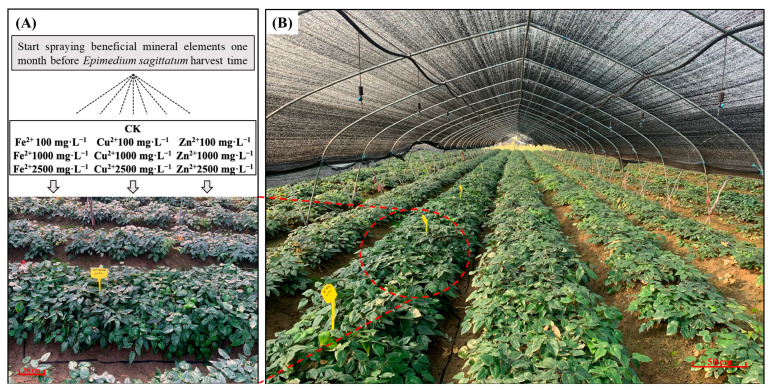
Overview of experimental sites used for spraying mineral elements on *E. sagittatum*. (**A**) Diagram of the spraying of mineral elements before *E. sagittatum* harvest. (**B**) The growth situation of *E. sagittatum* in special cultivation facilities.

Three-year-old *Epimedium sagittatum* experimental materials (Figure 1) were planted at the standardized planting base used for medicinal plants in Zhumadian City, Henan Province, PR China (33°0′ N, 114°29′ E). As *E. sagittatum* is a shade plant, all *E. sagittatum* plants were planted in special cultivation facilities with about 70~80% net complete shade. During the entire growth cycle of the *E. sagittatum* plants, workers at the standardized planting base followed the operational specifications for cultivation. The experiment started one month before the harvesting of *E. sagittatum* plants—the critical period for the accumulation of flavonoid bioactive compounds in *E. sagittatum* leaves. Aqueous Fe^2+^ solutions, aqueous Cu^2+^ solutions, and aqueous Zn^2+^ solutions of 100 mg·L^−1^, 1000 mg·L^−1^, and 2500 mg·L^−1^, respectively, were prepared for spraying on *E. sagittatum* leaves. Three different sulfates (FeSO_4_·7H_2_O, CuSO_4_·5H_2_O, and ZnSO_4_·7H_2_O) were purchased from Shanghai Aladdin Biochemical Technology Co., Ltd. To avoid the effects of the different degrees of hydration of different sulfates, we configured the aqueous solution with the mass concentration of metal ions (Fe^2+^, Cu^2+^ and Zn^2+^). Different concentrations of aqueous mineral element solutions were sprayed on the leaves of *E. sagittatum* in different experimental plots (three 2 m × 4 m areas per plot, arranged according to the randomized complete block design) for three consecutive days (at around 17:00 every day). Equal amounts of water were sprayed on the control group (CK), and the amounts of solution sprayed on each experimental plot were kept the same. The leaves of *E. sagittatum* were collected at 10 days, 20 days, and 30 days after the completion of the experimental treatments (CK, Fe^2+^ 100 mg·L^−1^, Fe^2+^ 1000 mg·L^−1^, Fe^2+^ 2500 mg·L^−1^, Cu^2+^ 100 mg·L^−1^, Cu^2+^ 1000 mg·L^−1^, Cu^2+^ 2500 mg·L^−1^, Zn^2+^ 100 mg·L^−1^, Zn^2+^ 1000 mg·L^−1^, and Zn^2+^ 2500 mg·L^−1^). The collected leaves of *E. sagittatum* were placed in an oven and dried at 50 °C. The dried leaves of *E. sagittatum* were crushed into powder and passed through a sieve (No. 3) to obtain samples for HPLC and FT-IR analysis.

### 2.2. Determination of the Four Flavonoids’ Contents in E. sagittatum Leaves by HPLC

The powdered sample (0.2 g) was accurately weighed and placed in a conical flask with a stopper. Then, 20 mL of aqueous ethanol solution (75:25, *v*/*v*) was added to the conical flask and weighed. The conical flask was shaken in an ultrasonic bath at 35 °C (power 400 W, frequency 50 KHz) for 30 s and then sonicated for 60 min. After cooling the conical flask, it was weighed, and the weight loss was replenished with ethanol aqueous solution (75:25, *v*/*v*). It was then shaken well and filtered through a 0.45 μm filter for HPLC analysis. The contents of icariin, epimedin A, epimedin B, and epimedin C in the sample solutions were determined using a Waters 2695 liquid chromatography system (Waters Corporation, Milford, MA, USA) equipped with an Agilent ZORBAX SB-C18 column (4.6 mm × 150 mm, 5 μm). Methanolic aqueous solutions containing icariin, epimedin A, epimedin B, and epimedin C (standards) were prepared and diluted to the appropriate concentrations to establish the calibration curves. The elution procedure, operating conditions, and calibration curves of the liquid chromatography system were as reported in our previous paper [22]. The chromatogram of the mixed standards and samples of *E. sagittatum* is shown in Figure S1. The sums of the main flavonoid contents in the samples were calculated based on the total amounts of icariin, epimedin A, epimedin B, and epimedin C determined by HPLC.

### 2.3. Establishment of FT-IR Spectra of E. sagittatum Leaf Samples

Sample preparation for FT-IR analysis: The powder of the sample (0.006 g) and the dried potassium bromide (0.6 g) were accurately weighed, respectively, and they were ground thoroughly in a natural agate mortar to obtain a mixed powder. The mixed powder (0.01 g) was accurately weighed, transferred to a press mold, spread evenly, and then pressed for 5 s at 10 MPa using a tablet press to obtain the sample to be tested. The samples to be tested were further selected for homogeneity and transparency, without obvious particles or broken cracks, for FT-IR analysis. Blank samples were made using unadulterated and dried potassium bromide following the above steps.

Operating conditions for sample scanning: The sample scanning was performed with a Fourier transform infrared spectrometer (INVENIO-S, Bruker, Bremen, Germany). The scanning resolution was 4 cm^−1^ with blank samples as the background, and the rates of sample scans and background scans were both 32 times/s. The scanning range was 4000~400 cm^−1^, and the interference of H_2_O and CO_2_ was deducted during the scanning. Five spectra were acquired for each sample.

### 2.4. Widely Untargeted Metabolomic Analysis of E. sagittatum Leaves

In order to further explain the effect of Fe^2+^ (2500 mg·L^−1^) treatment on the *E. sagittatum* leaves’ metabolite changes, we used a widely untargeted metabolomic to analyze the changes of metabolites in the Fe^2+^ treatment (*n* = 6) and CK (*n* = 6). This widely untargeted metabolomic was completed by LC-Bio Technologies (Hangzhou, China) Co., Ltd. The basic data and data identification results can be found in our previous papers [22]. This widely untargeted metabolomic was carried out on an ultra-performance liquid chromatography (UPLC)-MS/MS system connected to a high-resolution tandem mass spectrometer. A UPLC (UltiMate 3000 HPLC, Thermo Fisher Scientific, Waltham, MA, USA) and a Q-Exactive (Thermo Fisher Scientific, Waltham, MA, USA) made up the UPLC-MS/MS system. Metabolomics datasets were analyzed using the open source software metaX [23] (metaX provides several functions, such as peak picking and annotation, pathway annotation, correlation network analysis, and metabolite identification, and it is available at https://www.genomics.cn/ (accessed on 5 January 2023), and differential accumulation metabolites (DAMs) were obtained between Fe^2+^ and CK in *E. sagittatum* leaves.

### 2.5. Statistical Analysis

The HPLC data were processed and statistically analyzed using SPSS software (version 21.0, SPSS Inc., Chicago, IL, USA) by Duncan’s single-factor variance analysis and Pearson correlation analysis. The raw data of the FT-IR spectra were retrieved using OMNIC software (version 9.2, Nicolet, Madison, WI, USA), and orthogonal partial least squares discriminant analysis (OPLS-DA) of the characteristic absorption peaks of FT-IR spectra was performed using SIMCA-P software (version 14.1, Umetrics, Vasterbottens Lan, Sweden). The statistically analyzed data were graphically presented using the GraphPad Prism software (version 9.0, GraphPad Software Inc., San Diego, CA, USA).

## 3. Results

### 3.1. The Four Flavonoids’ Contents in E. sagittatum Leaves

**Figure 2:**
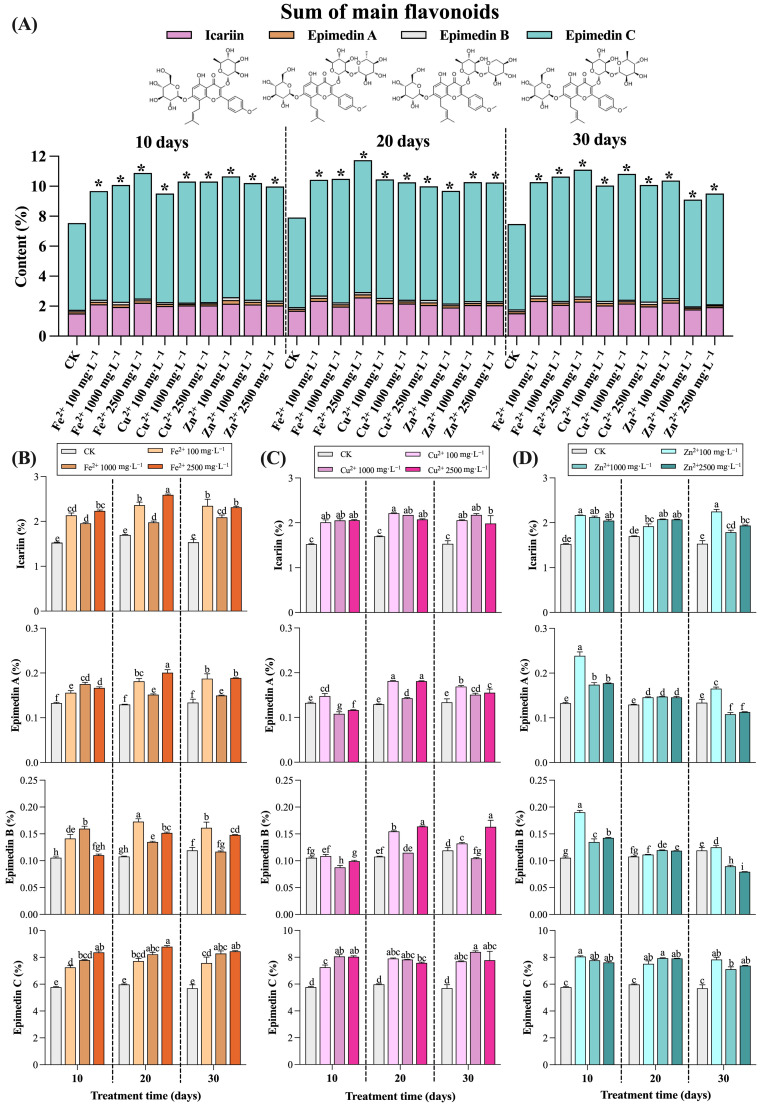
The changes of flavonoids in *E. sagittatum* leaves after spraying with mineral elements (10 days, 20 days, and 30 days). The data are expressed as the means ± SDs (standard deviations, *n* = 3). (**A**) The sum of main flavonoids in *E. sagittatum* leaves after spraying with Fe^2+^, Cu^2+^, and Zn^2+^ (10 days, 20 days, and 30 days). * indicates a significant difference between the treatment group and the control group. (**B**–**D**) The changes of icariin, epimedin A, epimedin B, and epimedin C in *E. sagittatum* leaves after spraying with Fe^2+^ (**B**), Cu^2+^ (**C**), and Zn^2+^ (**D**) (10 days, 20 days, and 30 days). The different lowercase letters indicate significant differences between treatments (*p* < 0.05) determined by Duncan’s single-factor variance analysis.

The sums of the main flavonoids’ contents, calculated by adding together the contents of icariin, epimedin A, epimedin B, and epimedin C in *E. sagittatum* leaves after 10 days, 20 days, and 30 days of treatment, are shown in Figure 2A. We observed that the sums of the main flavonoid contents in *E. sagittatum* leaves showed a significant increase at 10 days, 20 days, and 30 days after spraying with different concentrations of mineral elements. At 10 days after spraying with mineral elements, the contents of the main flavonoids in each treatment group ranged from 9.68% to 10.88%, which increased by 28.32% to 44.25% relative to CK (the sum of main flavonoids content in CK was 7.55%). At this time, the sum of the main flavonoids content in the treatment group (Fe^2+^ 2500 mg·L^−1^) was the highest, reaching 10.88%, and the sum of the main flavonoids content was significantly enhanced (*p* < 0.05). At 20 days after spraying with mineral elements, the contents of the main flavonoids in each treatment group ranged from 9.70% to 11.74%, which increased by 22.57% to 48.39% relative to CK (the sum of main flavonoids content in CK was 7.91%). At this time, the sum of the main flavonoids’ contents in the treatment group (Fe^2+^ 2500 mg·L^−1^) was the highest, reaching 11.74%, and the sum of the main flavonoids’ contents was significantly enhanced (*p* < 0.05). At 30 days after spraying with mineral elements, the content of the main flavonoids in each treatment group ranged from 9.11% to 11.10%, which increased by 21.75% to 48.40% relative to CK (the sum of main flavonoids content in CK was 7.48%). At this time, the sum of the main flavonoids’ contents in the treatment group (Fe^2+^ 2500 mg·L^−1^) was the highest, reaching 11.74%, and the sum of the main flavonoids’ contents was significantly enhanced (*p* < 0.05). The overall data we derived reflect that spraying with high concentrations of Fe^2+^ had the best effect in enhancing the sum of the main flavonoids’ contents in *E. sagittatum* leaves.

The effects of spraying Fe^2+^, Cu^2+^, and Zn^2+^ on the contents of the four flavonoid components (icariin, epimedin A, epimedin B, and epimedin C) in *E. sagittatum* leaves are presented in Figure 2B, Figure 2C, and Figure 2D, respectively. Figure 2B presents the changes in the contents of the four flavonoid components in *E. sagittatum* leaves at 10 days, 20 days, and 30 days after spraying with Fe^2+^ at low concentrations (100 mg·L^−1^), medium concentrations (1000 mg·L^−1^), and high concentrations (2500 mg·L^−1^). After spraying with high concentrations of Fe^2+^ (2500 mg·L^−1^), the contents of icariin, epimedin A, and epimedin C in *E. sagittatum* leaves reached their maximum values at 20 days, at 2.59%, 0.20%, and 8.80%, respectively, which increased by 52.77%, 54.74%, and 47.15%, respectively, relative to CK (*p* < 0.05). The content of epimedin B reached a maximum (0.17%) at 20 days after the low-concentration (100 mg·L^−1^) Fe^2+^ treatment, which increased by 60.07% compared to CK (*p* < 0.05).

Figure 2C presents the changes in the contents of the four flavonoid components in *E. sagittatum* leaves at 10 days, 20 days, and 30 days after spraying with Cu^2+^ at low concentrations (100 mg·L^−1^), medium concentrations (1000 mg·L^−1^), and high concentrations (2500 mg·L^−1^). The content of icariin reached a maximum (2.21%) at 20 days after the low concentration (100 mg·L^−1^) of Cu^2+^ treatment, which increased by 30.41% compared to CK (*p* < 0.05). The content of epimedin A reached a maximum (0.18%) at 20 days after the low-concentration (100 mg·L^−1^) and high-concentration (2500 mg·L^−1^) Cu^2+^ treatment, which increased by 39.63% compared to CK (*p* < 0.05). The content of epimedin B reached a maximum (0.16%) at 20 days and 30 days after the high-concentration (2500 mg·L^−1^) Cu^2+^ treatment, which increased by 51.80% compared to CK (*p* < 0.05). The content of epimedin C reached a maximum (8.39%) at 30 days after the medium concentration (1000 mg·L^−1^) of Cu^2+^ treatment, which increased by 47.34% compared to CK (*p* < 0.05).

Figure 2D presents the changes in the contents of the four flavonoid components in *E. sagittatum* leaves at 10 days, 20 days, and 30 days after spraying with Zn^2+^ at low concentrations (100 mg·L^−1^), medium concentrations (1000 mg·L^−1^), and high concentrations (2500 mg·L^−1^). The content of icariin reached a maximum (2.25%) at 30 days after the low concentration (100 mg·L^−1^) of Zn^2+^ treatment, which increased by 46.58% compared to CK (*p* < 0.05). After spraying with a low concentration of Zn^2+^ (100 mg·L^−1^), the contents of epimedin A, epimedin B, and epimedin C in *E. sagittatum* leaves reached their maximum values at 10 days, and at 0.24%, 0.19%, and 8.06%, respectively, which increased by 79.80%, 80.44%, and 39.40%, respectively, relative to CK (*p* < 0.05).

### 3.2. The FT-IR Spectra Characteristics in E. sagittatum Leaves

**Figure 3:**
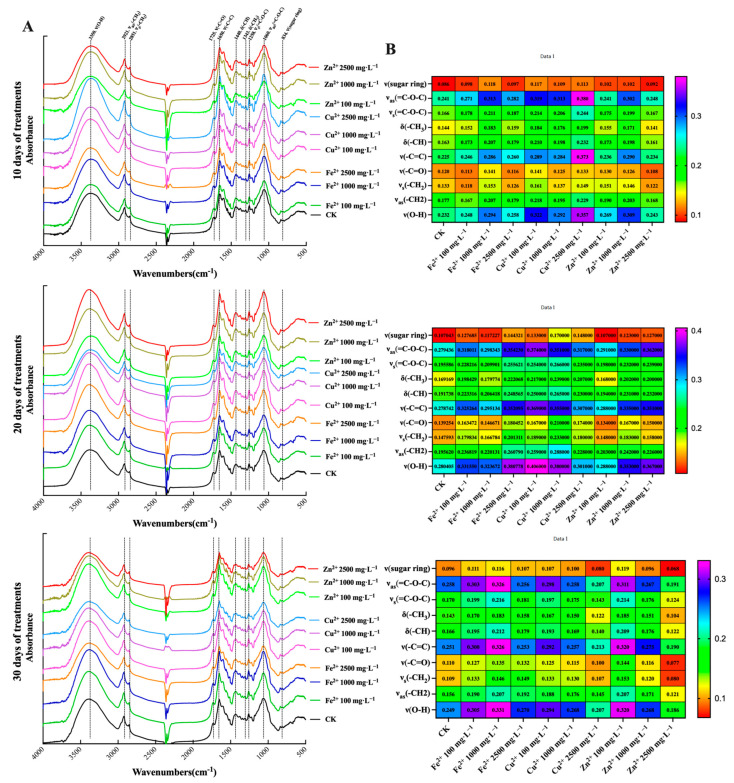
The characteristic absorption peaks of FT-IR spectra between 4000 and 500 cm^−1^ in *E. sagittatum* leaves after spraying with mineral elements (10 days, 20 days, and 30 days). The data are expressed as the means ± SDs (standard deviations, *n* = 5). (**A**) The FT-IR spectra between 4000 and 500 cm^−1^ in *E. sagittatum* leaves after 10 days, 20 days, and 30 days of treatment. (**B**) Heatmap of the changes of characteristic absorption peaks in *E. sagittatum* leaves after 10 days, 20 days, and 30 days of treatment.

The FT-IR spectra between 4000 and 500 cm^−1^ in *E. sagittatum* leaves at 10 days, 20 days, and 30 days after spraying with Fe^2+^, Cu^2+^, and Zn^2+^ at low concentrations (100 mg·L^−1^), medium concentrations (1000 mg·L^−1^), and high concentrations (2500 mg·L^−1^) are presented in Figure 3. As shown in the FT-IR spectra between 4000 and 500 cm^−1^ (Figure 3A), some peaks, including peak 3350, peak 2921, peak 2851, peak 1725, peak 1650, peak 1440, peak 1343, peak 1258, and peaks 1069 and 834 cm^−1^, emerged [24,25,26]. The assignment of functional groups responsible for IR absorption were carried out as follows: (1) absorption peak near 3350 cm^−1^, O–H stretching vibration, ν(O–H); (2) absorption peak near 2921 cm^−1^, –CH_2_ asymmetrical stretching vibration, ν_as_(–CH_2_); (3) absorption peak near 2851 cm^−1^, –CH_2_ symmetrical stretching vibration, ν_s_(–CH_2_); (4) absorption peak near 1725 cm^−1^, –C=O stretching vibration, ν(–C=O); (5) absorption peak near 1650 cm^−1^, –C=C stretching vibration, ν(–C=C); (6) absorption peak near 1440 cm^−1^, –CH bending vibration, δ(–CH); (7) absorption peak near 1343 cm^−1^, –CH_3_ bending vibration, δ(–CH_3_); (8) absorption peak near 1258 cm^−1^, =C–O–C symmetrical stretching vibration, ν_s_(=C–O–C); (9) absorption peak near 1069 cm^−1^, =C–O–C asymmetrical stretching vibration, ν_as_(=C–O–C); (10) absorption peak near 834 cm^−1^, sugar ring stretching vibration, ν(sugar ring). The above 10 characteristic absorption peaks in the FT-IR spectra of the *E. sagittatum* leaves changed significantly at 10 days, 20 days, and 30 days after treatment with different concentrations of mineral elements.

The absorbance variations of the above 10 characteristic absorption peaks at 10 days, 20 days, and 30 days after treatment with different concentrations of mineral elements are presented in Figure 3B. At 10 days after spraying with mineral elements, the absorbances of the peaks ν(O–H), ν(–C=C), and ν_as_(=C–O–C) were significantly up-regulated in treatment group Fe^2+^ 1000 mg·L^−1^, treatment group Cu^2+^ 100 mg·L^−1^, treatment group Cu^2+^ 1000 mg·L^−1^, treatment group Cu^2+^ 2500 mg·L^−1^, and treatment group Zn^2+^ 1000 mg·L^−1^. In treatment group Cu^2+^ 2500 mg·L^−1^, the absorbances of the peaks ν(O–H), ν(–C=C), and ν_as_(=C–O–C) were up-regulated from 0.232 to 0.357, from 0.225 to 0.373, and from 0.241 to 0.380, respectively. At 20 days after spraying with mineral elements, the absorbance changes of the peaks ν(O–H), ν(–C=C), and ν_as_(=C–O–C) were more significant. In treatment group Cu^2+^ 100 mg·L^−1^, the absorbances of the peaks ν(O–H), ν(–C=C), and ν_as_(=C–O–C) were up-regulated from 0.280 to 0.406, from 0.279 to 0.369, and from 0.279 to 0.374, respectively. At 30 days after spraying with mineral elements, the absorbance changes of the peaks ν(O–H), ν(–C=C), and ν_as_(=C–O–C) were more significant. In treatment group Fe^2+^ 1000 mg·L^−1^, the absorbances of the peaks ν(O–H), ν(–C=C), and ν_as_(=C–O–C) were up-regulated from 0.249 to 0.331, from 0.251 to 0.326, and from 0.258 to 0.326, respectively. Overall, the absorbances of the 10 characteristic peaks changed to different degrees with different treatment times and different concentrations of mineral elements.

### 3.3. The OPLS-DA Analysis of FT-IR Characteristic Spectra in E. sagittatum Leaves

**Figure 4:**
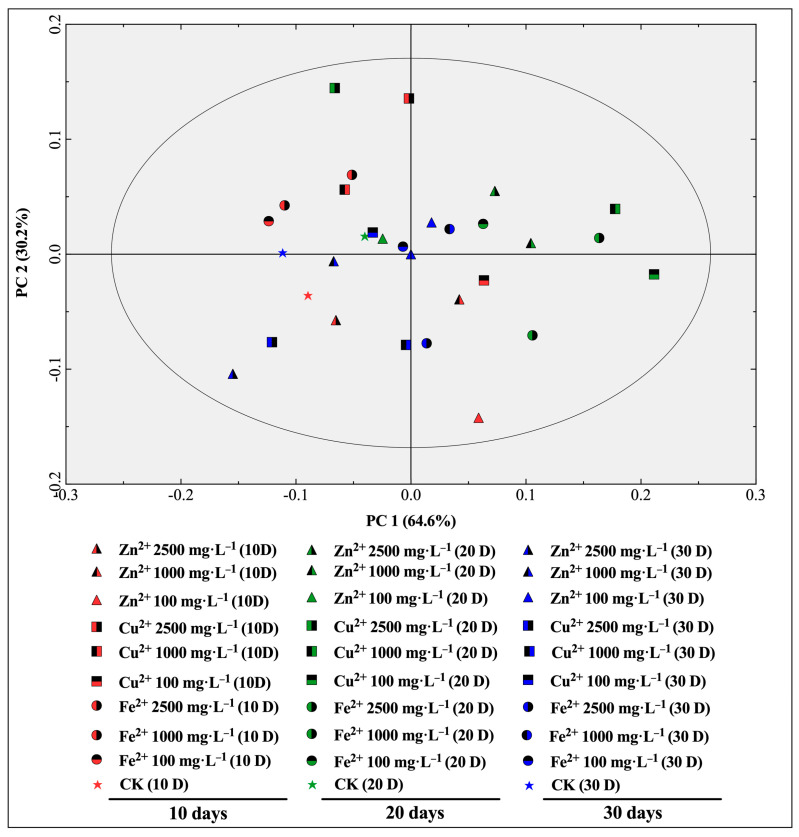
Scores scatter plot of the characteristic absorption peaks in *E. sagittatum* leaves subjected to FT-IR under the OPLS-DA model.

The OPLS-DA is a supervised statistical method used for discriminant analysis [27]. The relationship model between the samples subjected to different treatment times and different concentrations of mineral element treatments was established by focusing the classification of samples on principal components using OPLS-DA [28]. As shown in the score scatter plot of the characteristic absorption peaks in *E. sagittatum* leaves subjected to FT-IR under the OPLS-DA model (Figure 4), the first and second principal component axes (PC 1 and PC 2) explain a significant amount of the total variance (64.6% and 30.2%, respectively). The samples measured at 10 days after treatment are not distributed in the first quadrant, the samples 20 days after treatment are not distributed in the third quadrant, and the samples 30 days after treatment show a scattered distribution. Thus, the established OPLS-DA model provides better differentiation of the 30 samples with different treatment times and different concentrations of mineral elements. The differences in the samples can be identified via qualitative analysis using FT-IR characteristic absorption peaks.

**Figure 5:**
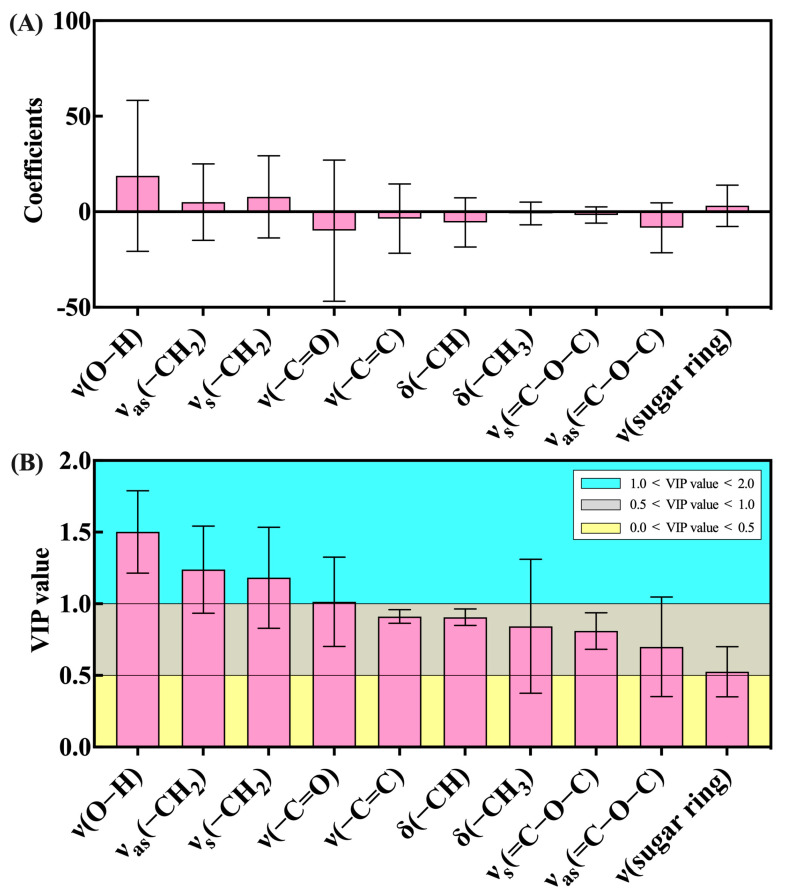
Coefficients plot (**A**) and VIP plot (**B**) of the characteristic absorption peaks in *E. sagittatum* leaves subjected to FT-IR under the OPLS-DA model.

The coefficients plot and variable importance for the projection (VIP) plot of the characteristic absorption peaks in *E. sagittatum* leaves subjected to FT-IR under the OPLS-DA model are presented by Figure 5. The coefficients plot (Figure 5A) indicates the degree of association of each X variable (absorbance of characteristic absorption peaks) in the system with the whole OPLS-DA model. The characteristic absorption peak ν(O–H) has the highest coefficient (18.81), followed by characteristic absorption peak ν(–C=O) (−9.82) and characteristic absorption peak ν_as_(=C–O–C) (−8.37). The VIP plot (Figure 5B) indicates the intensity and explanatory power of the impact of the differential characteristic absorption peaks on the classification discriminations of each group of samples. A VIP value >1 was used as the screening criterion to obtain the most important characteristic absorption peaks for the classification and discrimination of each group of samples, which were identified as peak ν(O–H), peak ν(–C=C), peak ν_as_(=C–O–C), and peak ν_as_(–CH_2_). The results show that the OPLS-DA model established in this study has good explanatory and reliable predictive power. Furthermore, the values of the coefficients and VIP can be used to determine the characteristic absorption peaks that contribute the most to the OPLS-DA model.

### 3.4. Widely Untargeted Metabolomic Analysis of E. sagittatum Leaves

**Figure 6:**
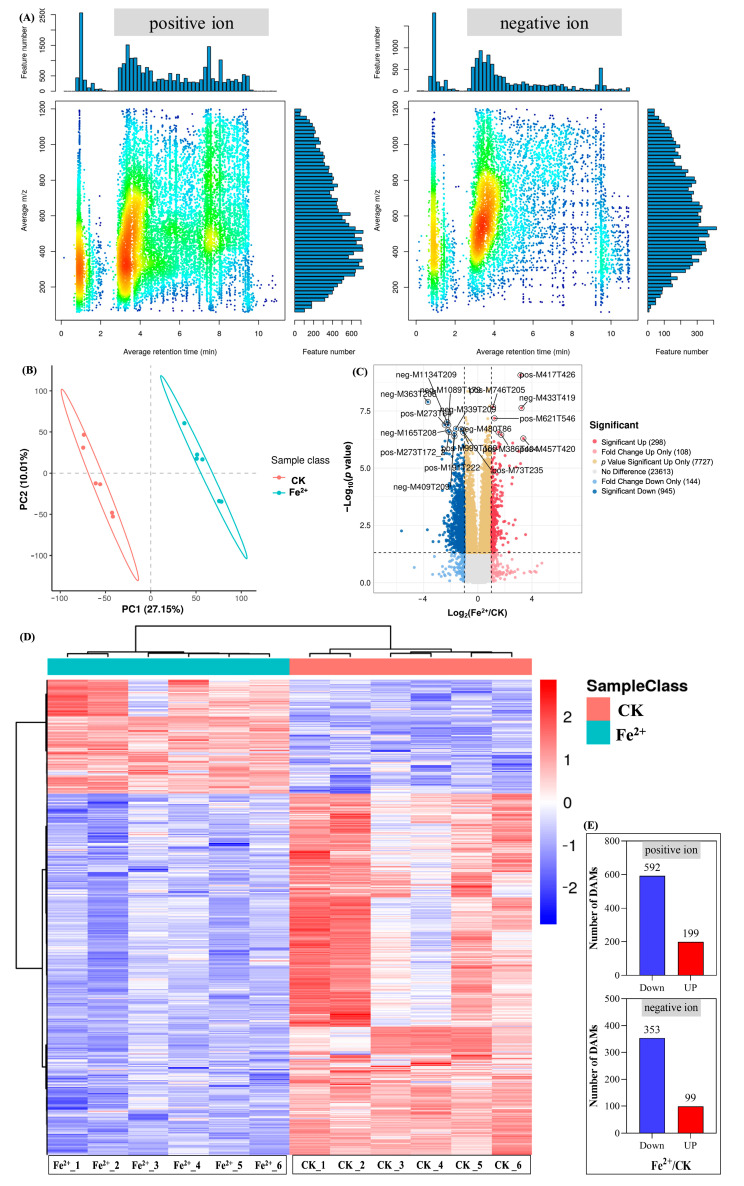
Basic metabolite information of Fe^2+^ (2500 mg·L^−1^) and CK based on widely untargeted metabolomics. (**A**) *M*/*z*-RT distribution of metabolites. (**B**) PLS-DA score plot of metabolites. (**C**) Significance analysis of the DAMs between the Fe^2+^ and CK determined by Volcano plot. (**D**) Fe^2+^ regulated the metabolites of *E. sagittatum* leaves. (**E**) Number of DAMs between Fe^2+^ and CK.

According to the HPLC analysis in this study, high-concentration Fe^2+^ (2500 mg·L^−1^) had a more positive effect on flavonoids in *E. sagittatum* leaves. Therefore, the widely untargeted metabolomic analysis carried out by UPLC-MS/MS aimed at deciphering the effects of Fe^2+^ on the metabolites in *E. sagittatum* leaves. Basic metabolite information of Fe^2+^ (2500 mg·L^−1^) and CK (*n* = 6) based on widely untargeted metabolomics is shown in Figure 6. The average retention time and *m*/*z* of positive and negative ions are shown in Figure 6A. The *m*/*z* of the positive ions is concentrated between 200 and 650. The *m*/*z* of the negative ions is concentrated between 400 and 800. We have demonstrated the overall differences in metabolites between Fe^2+^ and CK using the partial least squares discriminant analysis (PLS-DA) (Figure 6B), which effectively reveals the differences between the two groups of metabolites. Figure 6C–E show the significance analysis of DAMs between Fe^2+^ and CK. The Volcano plot also presents the most distinct DAMs between Fe^2+^ and CK. A total of 32,835 metabolites were detected in this study, of which 1243 metabolites showed significant differences (presented by Figure 6D), including 791 positive ions (199 significantly up-regulated DAMs and 592 significantly down-regulated DAMs) and 452 negative ions (99 significantly up-regulated DAMs and 353 significantly down-regulated DAMs). The next step of the analysis focused on the classes to which these DAMs belonged, and the patterns of change after high-concentration Fe^2+^ treatment.

**Figure 7:**
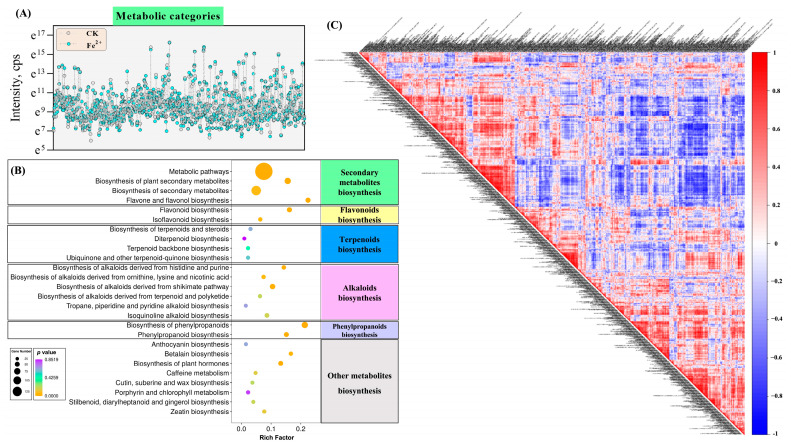
Identification and KEGG annotation of the DAMs between Fe^2+^ (2500 mg·L^−1^) and CK. (**A**) The abundances of DAMs belonging to different metabolic categories. (**B**) Statistic analysis of DAMs enriched in the secondary metabolites’ biosynthesis pathways by KEGG annotation. (**C**) Correlation between metabolites detected in the positive and negative ion mode (red—positive correlation, blue—negative correlation).

After the identification and KEGG annotation of DAMs, we screened for various metabolites involved in *E. sagittatum* leaves’ secondary metabolic activities, as shown in Figure 7. We calculated the normalized ionic intensities of each of the DAMs involved in different secondary metabolite activities (Figure 7A). The ionic intensities of DAMs changed significantly under high-concentration Fe^2+^ treatment. These DAMs were assigned to different classes of secondary metabolites, mainly including flavonoids, terpenoids, alkaloids, phenylpropanoids, and others. Figure 7B shows the statistical analysis of DAMs enriched in secondary metabolites’ biosynthesis pathways using KEGG annotation. It can be seen that the DAMs involved in different classes of secondary metabolites’ biosynthesis pathways exhibit distinct differences. Further, we performed a correlation analysis on the DAMs, as shown in Figure 7C, where red represents positive correlation and blue represents negative correlation, and the darker the color, the stronger the correlation between DAMs. It shows that there is an interaction between the changing DAMs in *E. sagittatum* leaves.

**Figure 8:**
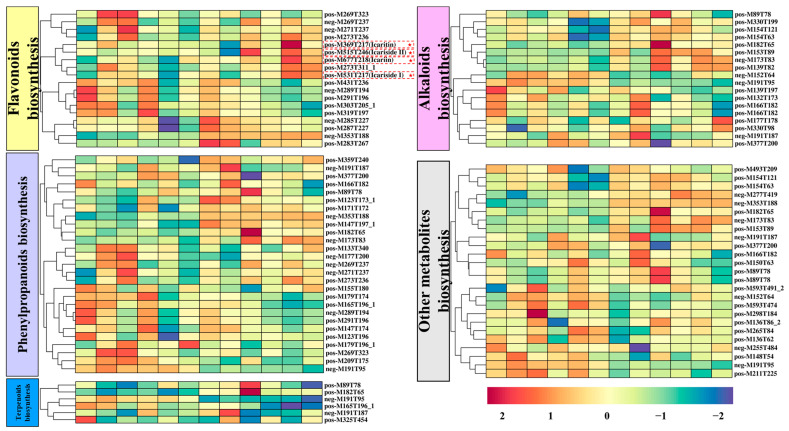
A heatmap of the relative amounts of flavonoids, phenylpropanoids, terpenoids, alkaloids, and other metabolites biosynthesis in *E. sagittatum* leaves.

The abundance of active secondary metabolites within the tissues of medicinal plants is a major focus of interest, and as an important herbal leafage, *E. sagittatum* is known for the richness of the flavonoid icariin in its leaves. In this study, we separately identified metabolites including flavonoids (18 metabolites), phenylpropanoids (27 metabolites), terpenoids (6 metabolites), alkaloids (18 metabolites), and others (25 metabolites) after high-concentration Fe^2+^ treatment (Figure 8). The abundant secondary metabolic components of *E. sagittatum* leaves showed rapid responses to high-Fe^2+^ treatment. The metabolites that were most up-regulated in the flavonoid biosynthesis pathway were neg-M353T188 (3-caffeoylquinic acid), neg-M285T227 (luteolin), and pos-M283T267 (pseudobaptigenin). More importantly, we also identified the characteristic active metabolites in *E. sagittatum*, including pos-M677T218 (icariin), pos-M369T217 (icaritin), pos-M531T217 (icariside I), and pos-M515T246 (icariside II). All four characteristic active metabolites showed different degrees of up-regulation after high-concentration Fe^2+^ treatment. The metabolites that were most up-regulated in the phenylpropanoid biosynthesis pathway were pos-M147T197_1 (coumarin), neg-M173T83 (shikimic acid), and pos-M182T65 (tyrosine). The metabolites that were most up-regulated in the alkaloid biosynthesis pathway were pos-M153T89 (xanthine), pos-M139T82 (3,4-dihydroxybenzaldehyde), and pos-M330T199 (reticuline). The above heatmap analysis shows that high-concentration Fe^2+^ treatment changed the flow of the secondary metabolism in *E. sagittatum* leaves, and stimulated the synthesis of different kinds of secondary metabolites, especially flavonoids (including the characteristic active metabolites icariin, icaritin, icariside I, and icariside II).

## 4. Discussion

Quality control during the harvesting period of *E. sagittatum* is necessary to ensure the stability and reliability of its herbal leafage [29]. In this study, we conducted an experiment on *E. sagittatum* leaves by spraying them with mineral elements, with the aim of improving the quality of the herbal leafage during the harvesting period. The results can provide a scientific basis for the improvement of agronomic cultivation measures during the harvesting period of *E. sagittatum*, and effectively improve the quality of *E. sagittatum* herbal leafage.

Flavonoids, including icariin, epimedin A, epimedin B, and epimedin C, are the main bioactive components of *E. sagittatum*, and are also the main indicators of interest for the quality control of *E. sagittatum* [2]. The harvesting period of herbal leafage is mainly concentrated in autumn every year, and this is the key point at which to control the stable and reliable quality of herbal leafage. The foliar spraying of mineral elements allows the mineral elements to enter the tissues directly from the plant leaves’ surfaces, so as to participate in the metabolism of plants and the synthesis of organic matter, which is faster and more effective than soil fertilization [30]. Some studies have reported that spraying mineral elements on leaves is an effective way to enhance the quality of medicinal herbs. The foliar application concentrations of 500 mg·L^−1^ of Fe^2+^, Mn^2+^, and Cu^2+^, and 5000 mg·L^−1^ of Zn^2+^ improved both *Panax ginseng* yield and ginsenosides content [31]. The foliar application of 3 g·L^−1^ ZnO can improve the yield, flower number, and crocin content of *Crocus sativus* [32]. The application of Fe + Mn + Zn each at 150 ppm, combined with 4 mL·L^−1^ seaweed extract, caused a significant increase in the essential oil percentage and artemisinin content in *Artemisia annua* [33]. We conducted a detailed experiment involving spraying different concentrations of elements (Fe^2+^, Cu^2+^, and Zn^2+^) on the leaves of *E. sagittatum* during the harvest period. Applying different concentrations of Fe, Cu, and Zn at different treatment times during the harvesting period of *E. sagittatum* could increase the sum of main flavonoids’ contents (*p* < 0.05). In particular, the content of main flavonoids reached a maximum at 20 days after the high concentration of Fe^2+^ (2500 mg·L^−1^) treatment and was elevated by 48.39% compared to CK (*p* < 0.05). This increase in the sum of main flavonoids’ contents in *E. sagittatum* leaves was mainly due to the increase in the contents of icariin and epimedin C. The contents of icariin and epimedin C in *E. sagittatum* leaves accounted for more than 95% of the sum of the main flavonoids. Mineral elements can act as catalysts for certain organic synthesis reactions in plants, or participate in the structural functions of plant bioactive components to influence the formation and accumulation of phytochemicals, thus ultimately affecting the quality of medicinal herbs via the bioactive components’ content [34]. As an essential element for chlorophyll synthesis, Fe is an important component and activator of many enzymes in plants [35], and is involved in redox and electron transfer, which directly or indirectly affects photosynthesis, respiration, and material–energy conversion [36]. Therefore, the scientific and reasonable supplementation of Fe fertilizer is an important way to alleviate reductions in medicinal plant yield and quality [37]. Our experimental results show that the leaf surface-spraying of mineral elements was an effective measure to enhance the flavonoids content in *E. sagittatum* during the harvest period; in particular, the highest total flavonoid content was reached after 20 days of high-Fe^2+^ (2500 mg·L^−1^) treatment. Thus, a higher-quality *E. sagittatum* herb can be obtained by means of the foliar application of Fe^2+^ before harvesting, which has practical significance as regards enhancing the economic value of cultivated *E. sagittatum*.

In addition to the accurate quantitative analysis of compounds, researchers are seeking non-destructive, rapid and accurate qualitative analysis tools for medicinal plants. FT-IR technology based on the Fourier transform of the interfering infrared light, with a Michael interferometer instead of a spectroscopic system, is characterized by fast scanning speed, high luminous flux, and a wide measurement range, and is by far the most commonly used method for infrared spectral analysis [25]. FT-IR technology determines the change in molecular coupling distance caused by the vibration or rotation of molecules [38]. Since the molecules of substances contain different functional groups and chemical bonds, or functional groups in different chemical environments, the energy required for vibrational energy level jumps is different, and the corresponding absorption of infrared light is also different, so the absorption peaks appear at different wavelengths, and therefore different and informative infrared spectra can be obtained [39]. The FT-IR technique was used to identify the absorption peaks of the functional groups of the samples, and enables fast and non-destructive identification of the contents of various chemical components of different kinds in medicinal plant samples [40]. Currently, FT-IR technology is widely used in the field of authenticity identification and chemical composition prediction for Chinese herbal medicines [41,42]. Yang et al. established a partial least squares regression model for different geographical regions of *Paris polyphylla* var. *yunnanensis* using the total flavonoid content and the specific absorption peaks identified by FT-IR of different samples [26], which distinguished the geographical origin and predicted the active ingredients of samples. Chen et al. successfully identified wild-type, cultivated-type, and tissue cultured-type *Anoectochilus roxburghii* by constructing a model involving the Tri-step identification method of infrared spectroscopy [43]. Chen et al. developed a correlation-based linear discrimination model for *Lonicera* plants using FT-IR, which can accurately distinguish the medicinal flower buds of five kinds of *Lonicera* plants [44]. In this study, we used FT-IR to identify 10 characteristic absorption peaks, including peaks ν(O–H), ν(–C=C) and ν_as_(=C–O–C), for 30 *E. sagittatum* leaf samples with different concentrations of mineral elements at different treatment times. The absorbances of the 10 characteristic peaks changed to significantly different degrees, and this better reflects the differences in the 30 *E. sagittatum* leaf samples. We also used OPLS-DA analysis to model the relationship between samples treated for different treatment times and with different concentrations of mineral elements. A total of 30 samples were well-distinguished in the score scatter plot; samples measured 10 days after treatment were not distributed in the first quadrant, samples 20 days after treatment were not distributed in the third quadrant, and samples 30 days after treatment showed a scattered distribution, indicating that the spraying of mineral elements did change the composition and content of chemical components in *E. sagittatum* leaves. Combined with the coefficients and VIP values of the 10 characteristic peaks in the OPLS-DA model, designation peak ν(O–H), peak ν(–C=C), peak ν_as_(=C–O–C), and peak ν_as_(–CH_2_) were shown to be relatively more important in sample differentiation and identification. Overall, we analyzed the FT-IR spectra characteristics of *E. sagittatum* leaf samples with different concentrations of mineral elements at different treatment times using the FT-IR technique and constructed an OPLS-DA model to achieve qualitative analysis and differentiated identification of *E. sagittatum* samples. This provides a feasible operational solution for the rapid and precise differentiation and identification of different samples of *E. sagittatum*, and contributes to our understanding of the effect of spraying mineral elements on the changes in chemical composition of *E. sagittatum* leaves.

Metabolomics is an emerging histological technology that is a dynamic product of the processes and activities of states of life, and can be used to determine physiological changes based on variability [45]. At present, metabolomics has been applied in many industries and fields, including traditional Chinese medicine [46]. In the study of medicinal plants, the main focus is on the identification of the basal origin, cultivation, harvesting, processing, concoction, and pharmacology [47]. Metabolomics is mainly divided into targeted and untargeted metabolomics. Because of its ability to analyze different classes of metabolites, untargeted metabolomics have been widely used to compare the overall metabolic compositions of different samples [48]. Untargeted metabolomics allow for the quantitative analysis of all metabolites in an organism, and thus visualizes the changes occurring in the plant tissues, which is more convenient compared to other histological techniques [49]. The synthesis of active secondary metabolites in medicinal plants is affected by environmental factors such as temperature, light, and moisture in different regions, and the variability of harvesting time and the complexity of active metabolites of different medicinal plants makes it difficult to study the formation of secondary metabolism [13,50]. Metabolomics technology plays an important role in the study of the secondary metabolism formation of medicinal plants given its high throughput and other technical analysis features. In this study, the effect of high-Fe^2+^ treatment on the synthesis of secondary metabolites (especially characteristic flavonoids) in *E. sagittatum* leaves during harvesting was investigated using widely untargeted metabolomics. After filtering, identification, and KEGG annotation, the high-quality metabolites were assigned to different secondary metabolism categories, including primarily flavonoids (18 metabolites), phenylpropanoids (27 metabolites), terpenoids (6 metabolites), alkaloids (18 metabolites), and others (25 metabolites). As the most important characteristic flavonoids, icariin, icaritin, icariside I, and icariside II were also annotated in the widely untargeted metabolomic analysis. The relative contents of these four metabolites were significantly up-regulated by the high-concentration Fe^2+^ treatment. This is consistent with the finding that high-concentration Fe^2+^ treatment increased the content of icariin in HPLC analysis. It is shown that widely untargeted metabolomics is one of the most effective techniques in explaining the secondary metabolic synthesis of medicinal plants. In summary, our study further confirms the positive effect of high-Fe^2+^ treatment on secondary metabolic activities in *E. sagittatum* leaves during the harvesting period by widely untargeted metabolomic analysis.

## 5. Conclusions

In this study, we conducted an experiment on *E. sagittatum* leaves sprayed with mineral elements, with the aim of improving the quality of the herbal leafage during the harvesting period. We elucidated the changes in flavonoids (icariin, epimedin A, epimedin B, and epimedin C) in *E. sagittatum* leaves via quantitative analysis. The content of the main flavonoids reached a maximum (11.74%) at 20 days after the high concentration of Fe^2+^ (2500 mg·L^−1^) treatment. From the perspective of the qualitative analysis, we analyzed the FT-IR spectra characteristics of *E. sagittatum* leaf samples using the FT-IR technique, and constructed an OPLS-DA model, then identified several key characteristic peaks to complete the differentiated identification of *E. sagittatum* samples. Further, widely untargeted metabolomic analysis identified different classes of metabolites, including flavonoids (18 metabolites), phenylpropanoids (27 metabolites), terpenoids (6 metabolites), alkaloids (18 metabolites), and others (25 metabolites). As the most important characteristic flavonoids, the relative contents of icariin, icaritin, icariside I, and icariside II were found to be up-regulated by high-Fe^2+^ treatment. Our experimental results demonstrate that high-concentration Fe^2+^ treatment is an effective measure to increase the flavonoids content in *E. sagittatum* leaves during the harvesting period, which can provide a scientific basis for the improvement of *E. sagittatum* leaf cultivation agronomic measures.

## Data Availability

The data presented in this study are available on request from the corresponding author. The data are not publicly available due to their required use in further research.

## Supplementary Materials

The following supporting information can be downloaded at: https://www.mdpi.com/article/10.3390/metabo13020294/s1, Figure S1: The chromatogram of the mixed standards and samples of *E. sagittatum*.

## References

[1] Chen X.-J., Tang Z.-H., Li X.-W., Xie C.-X., Lu J.-J., Wang Y.-T. (2015). Chemical constituents, quality control, and bioactivity of *Epimedii Folium* (Yinyanghuo). Am. J. Chin. Med..

[2] Ma H., He X., Yang Y., Li M., Hao D., Jia Z. (2011). The genus *Epimedium*: An ethnopharmacological and phytochemical review. J. Ethnopharmacol..

[3] Chen J., Xu Y., Wei G., Liao S., Zhang Y., Huang W., Yuan L., Wang Y. (2015). Chemotypic and genetic diversity in *Epimedium sagittatum* from different geographical regions of China. Phytochemistry.

[4] Zhang Y., Yang L., Chen J., Sun W., Wang Y. (2014). Taxonomic and phylogenetic analysis of *Epimedium* L. based on amplified fragment length polymorphisms. Sci. Hortic..

[5] Li W.-X., Deng Y.-Y., Li F., Liu B., Liu H.-Y., Shi J.-S., Gong Q.-H. (2015). Icariin, a major constituent of flavonoids from Epimedium brevicornum, protects against cognitive deficits induced by chronic brain hypoperfusion via its anti-amyloidogenic effect in rats. Pharmacol. Biochem. Behav..

[6] Wu H., Lien E.J., Lien L.L., Wu H., Lien E.J., Lien L.L., Schultz R.M., Ram V.J., Domingo E., Spence P., Gupta S.P., Bhat S.P., Villarreal E.C. (2003). Chemical and pharmacological investigations of *Epimedium* species: A survey. Progress in Drug Research.

[7] Jiang J., Song J., Jia X.-B. (2015). Phytochemistry and ethnopharmacology of *Epimedium* L. Species. Chin. Herb. Med..

[8] Zhang H.-F., Zhang X., Yang X.-H., Qiu N.-X., Wang Y., Wang Z.-Z. (2013). Microwave assisted extraction of flavonoids from cultivated *Epimedium sagittatum*: Extraction yield and mechanism, antioxidant activity and chemical composition. Ind. Crops Prod..

[9] Shen P., Guo B.L., Gong Y., Hong D.Y.Q., Hong Y., Yong E.L. (2007). Taxonomic, genetic, chemical and estrogenic characteristics of *Epimedium* species. Phytochemistry.

[10] Bi Z., Zhang W., Yan X. (2022). Anti-inflammatory and immunoregulatory effects of icariin and icaritin. Biomed. Pharmacother..

[11] Sun Y., Li Q., Xu J.-M., Liang J., Cheng Y., Li S., Zheng L., Ye B., Meng K., Qin S. (2018). A multicenter, single arm phase II trial of a small molecule immune-modulator icaritin: Safety, overall survival, immune dynamics, and PD-L1 expression in advanced hepatocellular carcinoma. J. Clin. Oncol..

[12] Mo D., Zhu H., Wang J., Hao H., Guo Y., Wang J., Han X., Zou L., Li Z., Yao H. (2021). Icaritin inhibits PD-L1 expression by Targeting Protein IκB Kinase α. Eur. J. Immunol..

[13] Li Y., Kong D., Fu Y., Sussman M.R., Wu H. (2020). The effect of developmental and environmental factors on secondary metabolites in medicinal plants. Plant Physiol. Biochem..

[14] Alaerts G., Pieters S., Logie H., Van Erps J., Merino-Arévalo M., Dejaegher B., Smeyers-Verbeke J., Vander Heyden Y. (2014). Exploration and classification of chromatographic fingerprints as additional tool for identification and quality control of several *Artemisia* species. J. Pharm. Biomed. Anal..

[15] Gomes A.F., Almeida M.P., Leite M.F., Schwaiger S., Stuppner H., Halabalaki M., Amaral J.G., David J.M. (2019). Seasonal variation in the chemical composition of two chemotypes of *Lippia alba*. Food Chem..

[16] Huang W., Zeng S., Xiao G., Wei G., Liao S., Chen J., Sun W., Lv H., Wang Y. (2015). Elucidating the biosynthetic and regulatory mechanisms of flavonoid-derived bioactive components in *Epimedium sagittatum*. Front. Plant Sci..

[17] Tripathi P., Singh A., Rakshit A., Meena V.S., Parihar M., Singh H.B., Singh A.K. (2021). Chapter 19—Biofertilizers: “An ace in the hole” in medicinal and aromatic plants cultivation. Biofertilizers.

[18] Toksha B., Sonawale V.A.M., Vanarase A., Bornare D., Tonde S., Hazra C., Kundu D., Satdive A., Tayde S., Chatterjee A. (2021). Nanofertilizers: A review on synthesis and impact of their use on crop yield and environment. Environ. Technol. Innov..

[19] Bayram S.E., İsfendiyaroğlu M., Tuncay Ö. (2022). Effects of foliar zinc applications on some yield parameters and essential oil constituents of the mastic tree (*Pistacia lentiscus* var. *chia* Duham.). J. Appl. Res. Med. Aromat. Plants.

[20] He Y., Luo Y., Wang Q., Sun Y., Duan N., Chen Z., Zeng H. (2021). Spray treatment of leaves with Fe^2+^ promotes procyanidin biosynthesis by upregulating the expression of the F3H and ANS genes in red rice grains (*Oryza sativa* L.). J. Cereal Sci..

[21] Chrysargyris A., Papakyriakou E., Petropoulos S.A., Tzortzakis N. (2019). The combined and single effect of salinity and copper stress on growth and quality of *Mentha spicata* plants. J. Hazard. Mater..

[22] Yang L., Zhou S., Hou Y., Ji B., Pei L., Su X., Zhong H., Dong C. (2022). Blue light induces biosynthesis of flavonoids in *Epimedium sagittatum* (Sieb.et Zucc.) Maxim. leaves, a study on a light-demanding medicinal shade herb. Ind. Crops Prod..

[23] Wen B., Mei Z., Zeng C., Liu S. (2017). metaX: A flexible and comprehensive software for processing metabolomics data. BMC Bioinform..

[24] Pei L.-K., Sun S.-Q., Guo B.-L., Huang W.-H., Xiao P.-G. (2008). Fast quality control of Herba *Epimedii* by using Fourier transform infrared spectroscopy. Spectrochim. Acta Part A Mol. Biomol. Spectrosc..

[25] Krysa M., Szymańska-Chargot M., Zdunek A. (2022). FT-IR and FT-Raman fingerprints of flavonoids—A review. Food Chem..

[26] Yang Y., Zhao Y., Zuo Z., Wang Y. (2019). Determination of total flavonoids for *Paris polyphylla* var. *yunnanensis* in different geographical origins using UV and FT-IR spectroscopy. J. AOAC Int..

[27] Boccard J., Rutledge D.N. (2013). A consensus orthogonal partial least squares discriminant analysis (OPLS-DA) strategy for multiblock Omics data fusion. Anal. Chim. Acta.

[28] Huang B.-M., Zha Q.-L., Chen T.-B., Xiao S.-Y., Xie Y., Luo P., Wang Y.-P., Liu L., Zhou H. (2018). Discovery of markers for discriminating the age of cultivated ginseng by using UHPLC-QTOF/MS coupled with OPLS-DA. Phytomedicine.

[29] Singh P.A., Bajwa N., Chinnam S., Chandan A., Baldi A. (2022). An overview of some important deliberations to promote medicinal plants cultivation. J. Appl. Res. Med. Aromat. Plants.

[30] Kumar D., Punetha A., Verma P.P.S., Padalia R.C. (2022). Micronutrient based approach to increase yield and quality of essential oil in aromatic crops. J. Appl. Res. Med. Aromat. Plants.

[31] Zhang H., Yang H., Wang Y., Gao Y., Zhang L. (2013). The response of ginseng grown on farmland to foliar-applied iron, zinc, manganese and copper. Ind. Crops Prod..

[32] Rostami M., Talarposhti R.M., Mohammadi H., Demyan M.S. (2019). Morpho-physiological Response of Saffron (*Crocus sativus* L.) to Particle Size and Rates of Zinc Fertilizer. Commun. Soil Sci. Plant Anal..

[33] Ghatas Y., Ali M., Elsadek M., Mohamed Y. (2021). Enhancing growth, productivity and artemisinin content of *Artemisia annua* L. Plant using seaweed extract and micronutrients. Ind. Crops Prod..

[34] Awasthi S., Chauhan R., Srivastava S., Kumar V., Srivastava A.K., Suprasanna P. (2022). Chapter 2—The importance of beneficial and essential trace and ultratrace elements in plant nutrition, growth, and stress tolerance. Plant Nutrition and Food Security in the Era of Climate Change.

[35] Li P., Wang A., Du W., Mao L., Wei Z., Wang S., Yuan H., Ji R., Zhao L. (2020). Insight into the interaction between Fe-based nanomaterials and maize (*Zea mays*) plants at metabolic level. Sci. Total Environ..

[36] Liang G. (2022). Iron uptake, signaling, and sensing in plants. Plant Commun..

[37] Afzal S., Singh N.K., Singh N., Sohrab S., Rani M., Mishra S.K., Agarwal S.C., Aftab T., Hakeem K. (2022). Chapter 9—Effect of metals and metalloids on the physiology and biochemistry of medicinal and aquatic plants. Metals Metalloids Soil Plant Water Systems.

[38] Esteki M., Memarbashi N., Simal-Gandara J. (2022). Classification and authentication of tea according to their harvest season based on FT-IR fingerprinting using pattern recognition methods. J. Food Compos. Anal..

[39] Nikalje G.C., Kumar J., Nikam T.D., Suprasanna P. (2019). FT-IR profiling reveals differential response of roots and leaves to salt stress in a halophyte *Sesuvium portulacastrum* (L.). Biotechnol. Rep..

[40] He W., Lei T. (2020). Identification of camellia oil using FT-IR spectroscopy and chemometrics based on both isolated unsaponifiables and vegetable oils. Spectrochim. Acta Part A Mol. Biomol. Spectrosc..

[41] Cheng C.-S., Wang C.-J., Liang J., Lao C.-C., Zhou H., Zhang Z.-F. (2017). A new approach for identification of medicinal almonds by fourier transform infrared spectroscopy and systematic clustering of characteristic peaks. Chin. J. Nat. Med..

[42] Shannon M., Lafeuille J.-L., Frégière-Salomon A., Lefevre S., Galvin-King P., Haughey S.A., Burns D.T., Shen X., Kapil A., McGrath T.F. (2022). The detection and determination of adulterants in turmeric using fourier-transform infrared (FTIR) spectroscopy coupled to chemometric analysis and micro-FTIR imaging. Food Control..

[43] Chen Y., Huang J., Yeap Z.Q., Zhang X., Wu S., Ng C.H., Yam M.F. (2018). Rapid authentication and identification of different types of *A. roxburghii* by Tri-step FT-IR spectroscopy. Spectrochim. Acta Part A Mol. Biomol. Spectrosc..

[44] Chen J., Guo B., Yan R., Sun S., Zhou Q. (2017). Rapid and automatic chemical identification of the medicinal flower buds of *Lonicera* plants by the benchtop and hand-held Fourier transform infrared spectroscopy. Spectrochim. Acta Part A Mol. Biomol. Spectrosc..

[45] Rinschen M.M., Ivanisevic J., Giera M., Siuzdak G. (2019). Identification of bioactive metabolites using activity metabolomics. Nat. Rev. Mol. Cell Biol..

[46] Yang L., Wen K.-S., Ruan X., Zhao Y.-X., Wei F., Wang Q. (2018). Response of plant secondary metabolites to environmental factors. Molecules.

[47] Zhou X., Li C.-G., Chang D., Bensoussan A. (2019). Current status and major challenges to the safety and efficacy presented by Chinese herbal medicine. Medicines.

[48] Scossa F., Benina M., Alseekh S., Zhang Y., Fernie A.R. (2018). The integration of metabolomics and next-generation sequencing data to elucidate the pathways of natural product metabolism in medicinal plants. Planta Med..

[49] Hong J., Yang L., Zhang D., Shi J. (2016). Plant metabolomics: An indispensable system biology tool for plant science. Int. J. Mol. Sci..

[50] Soni U., Brar S., Gauttam V.K. (2015). Effect of seasonal variation on secondary metabolites of medicinal plants. Int. J. Pharm. Sci. Res..

