# Supplementary figures and images for: A Comprehensive Analysis to Elucidate the Effects of Spraying Mineral Elements on the Accumulation of Flavonoids in Epimedium sagittatum during the Harvesting Period
Source: Metabolites. 2023 Feb 16;13(2):294. doi: 10.3390/metabo13020294 (PMC9964673; doi:10.3390/metabo13020294)

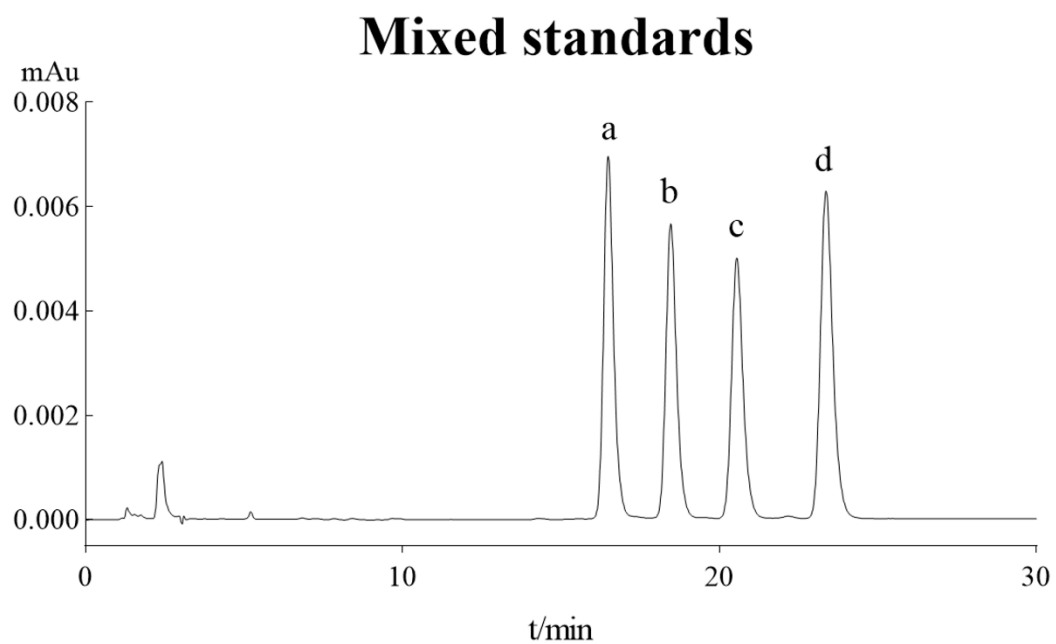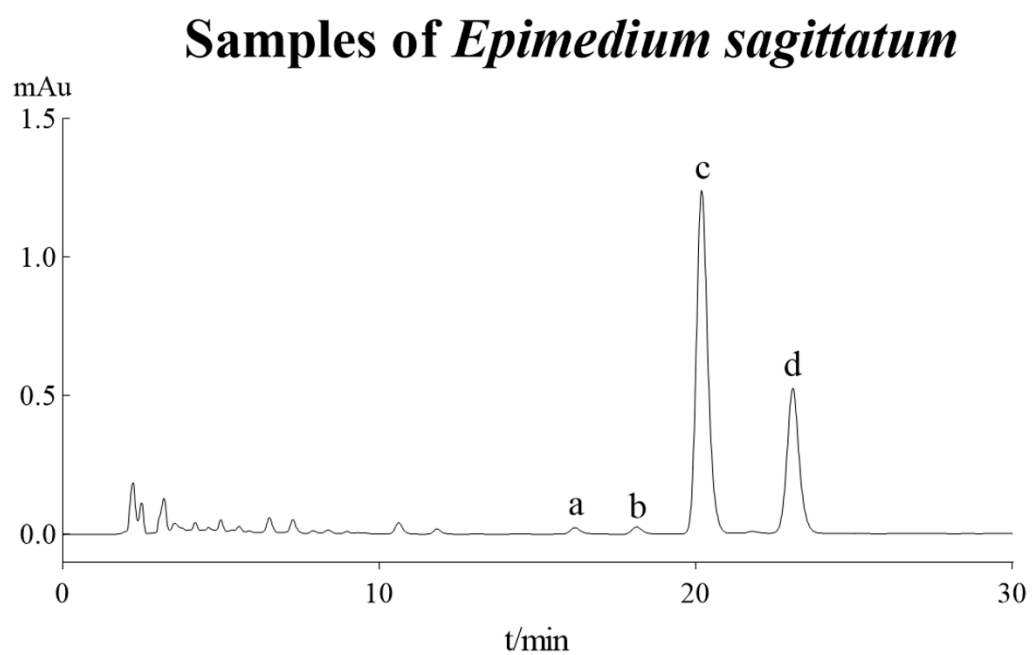

Figure S1. The chromatogram of the mixed standards and samples of *E. sagittatum*.

Supplement: Supplementary file 1 [file metabolites-13-00294-s001.zip › metabolites-2181102-supplementary.pdf]
